# Salvianolic Acid B Restored Impaired Barrier Function via Downregulation of MLCK by microRNA-1 in Rat Colitis Model

**DOI:** 10.3389/fphar.2016.00134

**Published:** 2016-05-26

**Authors:** Yongjian Xiong, Jingyu Wang, Hongwei Chu, Dapeng Chen, Huishu Guo

**Affiliations:** ^1^Central Laboratory, The First Affiliated Hospital, Dalian Medical UniversityDalian, China; ^2^Laboratory Animal Center, Dalian Medical UniversityDalian, China; ^3^Institute for Brain Disorder, College of Basic Medical Sciences, Dalian Medical UniversityDalian, China

**Keywords:** salvianolic acid B, inflammatory bowel disease, barrier function, microRNA-1, myosin light chain kinase

## Abstract

Salvianolic acid B (Sal B) is isolated from the traditional Chinese medical herb Salvia miltiorrhiza and is reported to have a wide range of therapeutic benefits. The aim of this study was to investigate the effects of Sal B on epithelial barrier dysfunction in rat colitis and to uncover related mechanisms. Rat colitis model was established by intracolonic administration of 2, 4, 6-trinitrobenzene sulfonic acid (TNBS). The intestinal barrier function was evaluated by measuring the serum recovery of fluorescein isothiocyanate-4 kD dextran *in vivo* and transepithelial electrical resistance *in vitro* respectively. The protein expression related to intestinal barrier function was studied using western blotting. The effects of Sal B on inflammatory responses, oxidative damage and colitis recurrence were also studied in this study. The intestinal barrier dysfunction in colitis was reversed by Sal B, accompanied with the decrease of tight junction proteins, and the effect could be blocked by microRNA-1(miR-1) inhibition. The inflammatory responses, oxidative damage and colitis recurrence were also decreased by Sal B. The colitis symptoms and recurrences were ameliorated by Sal B, and restoration of impaired barrier function via downregulation of MLCK by miR-1 maybe involved in this effect. This study provides some novel insights into both of the pathological mechanisms and treatment alternatives of inflammatory bowel disease.

## Introduction

Inflammatory bowel disease (IBD) is complex multi-factorial inflammatory diseases, of which Crohn's disease (CD) and ulcerative colitis (UC) are the two most common forms (Garcia de Tena et al., 2002). IBD are important diseases of the gastrointestinal tract and are associated with a high degree of patient impairment and treatment cost.

The pathogenesis of IBD is not fully understood at present, but it has been reported that the development of IBD is thought to be related to a combination of genetic and environmental factors (Matricon et al., 2010). Role of free radicals and epithelial barrier functions has attracted much attention in the pathogenesis of IBD recently (Almenier et al., 2012). As is known that abnormal mucosal immune reactions to lumenal content is a course of tissue damage in IBD (Simmonds et al., 1992). Infiltration of inflammatory cells into the mucosa and release of free radicals, such as reactive oxygen species (ROS) and reactive nitrogen species, could lead to inflammatory cascade (Almenier et al., 2012; Nagib et al., 2013). Tissue damage and additional recruitment of inflammatory cells could be caused by these free radicals, which then sustain the inflammatory cascade (Keshavarzian et al., 2003; Reuter et al., 2010; Almenier et al., 2012).

IBD could be evoked or aggravated by defective intestinal barrier function and the characteristics of epithelial tight junction defines intestinal barrier function with intact epithelium (Su et al., 2009, 2013; Al-Sadi et al., 2013) Restoration of barrier function maybe a promising way to alleviate IBD (Clayburgh et al., 2004). The high expression of myosin light chain kinase (MLCK), which induces contraction of the perijunctional actomyosin ring through myosin light chain phosphorylation, plays an important role in the increase of intestinal permeability and epithelial barrier dysfunction (Cunningham and Turner, 2012; Al-Sadi et al., 2013). MLCK regulation in epithelial barrier dysfunction may provide future therapeutic targets to treat intestinal diseases.

Micro RNAs (miRNAs) is a series of small non-coding RNA molecules (containing 20–25 nucleotides) found in eukaryotes, functioning in RNA silencing, post-transcriptional, and translation regulation of gene expression (Ambros, 2004; Bartel, 2004). Aberrant expression of miRNAs is involved in the pathophysiology of human diseases and it have been proved to be novel therapeutic targets for inflammation, cancer, and cardiovascular diseases (Chen, 2007; Bogatcheva et al., 2011). MicroRNA-1(miR-1) was shown to protect endothelial permeability through inhibition of myosin light chain kinase in cardiovascular disease (Hua et al., 2013). Previous studies showed that more and more polyphenols possess beneficial effects on human health through targeting of miRNA (Milenkovic et al., 2013; Tomé-Carneiro et al., 2013). Therefore, miR-1 may alleviate IBD symptoms through restoring the impaired barrier function. Our previous results showed that Sal B treatment affected the expression of miR-1 *in vitro*. In the present study, we determine whether Sal B could restore the impaired barrier function in rat colitis model through modulation of miR-1.

Salvia miltiorrhiza, also known as Danshen, has been widely used for thousands of years in traditional Chinese medicine, with little reported toxicity (Kim et al., 2005). As one of the major water soluble components of *Radix Salvia miltiorrhiza*, salvianolic acid B (Sal B) was shown to exert antioxidant activity (Li et al., 2015) and our pre-experiments also showed that Sal B restored abnormal intestinal permeability in Caco-2 cells. Sal B has been reported to possess a wide range of biological activities including anti-inflammatory, anti-oxidant, and anti-carcinogenic effects (Yao et al., 2009; Li et al., 2014). Colitis model was established by intracolonic administration of 2,4,6-trinitrobenzene sulfonic acid (TNBS), which is classic model for studying barrier function in IBD (Turner, 2009; Morampudi et al., 2014). Severe colonic damage, which is associated with high myeloperoxidase (MPO) activity and is mainly known as a reflection of neutrophilic infiltration into the damaged tissue, could be induced by TNBS dissolved in ethanol (Veljaca et al., 1995). The effects of Sal B on colitis were evaluated from the decrease of epithelial permeability and free radical scavenging, respectively. The effects of Sal B on the recurrence of colitis induced by interleukin-1 beta (IL-1β) were also investigated based on its protective effects on epithelial barrier function.

## Materials and methods

### Animals

Sprague-Dawley male rats (5–7 weeks of age, weighing 140–160 g) were purchased from the Experimental Animal Center, Dalian Medical University [Certificate of Conformity: No. SYXK (Liao) 2013–0006]. This study was carried out in accordance with “the recommendations of the National Institutes of Health Guide for Care and Use of Laboratory Animals (Publication no. 85–23, revised 1985) and Dalian Medical University Animal Care and Ethics Committee.” The experimental protocol was approved by “Dalian Medical University Animal Care and Ethics Committee” at Dec. 8th, 2015. The animal protocol was designed to minimize pain or discomfort to the animals. The animals were acclimatized to laboratory conditions (23°C, 12/12 h light/dark, 50% humidity, *ad libitum* access to food and water) for 2 weeks prior to experimentation and no animals died before experiments.

### Reagents

Sal B (Purify speciation: ≥98%) was obtained from the Chinese National Institute for the Control of Pharmaceutical and Biological Products (Beijing, China). Sulfasalazine (SASP) was purchased from Tianjin Kingyork Group Co. Ltd. (Tianjin, China). The NADPH oxidase 4 (NOX4) antibodies, superoxide dismutase 2 (SOD2) antibodies, malondialdehyde 5 (MDA 5) antibodies, glutathione 2 (GSH 2) antibodies, MLCK antibodies, ZO-1 antibodies, and occludin antibodies were obtained from Abcam (Hong Kong) Ltd. (Hong Kong, China); iNOS antibodies were obtained from Beijing Biosynthesis Biotechnology Co. Ltd (Beijing, China). Claudin-2 antibodies were obtained from Life Technologies (Carlsbad, CA, USA). Other agents were purchased from Sigma-Aldrich (Saint Louis, MO, USA).

### Experimental design

**Figure 1:**
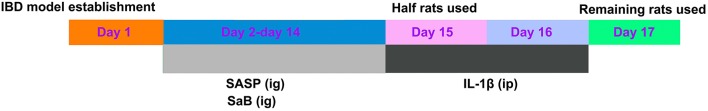
**Experimental flowchart for the present study**.

One hundred rats were randomly divided into five groups, each consisting of 20 rats. Group I served as sham operation group with intracolonic administration of equal volume of ethanol, then receive the gavage treatment of PBS. Groups II, III, IV, V were subjected for the induction of colitis. Group II served as the TNBS colitis group (TNBS) and groups III, IV, and V received treatment of SASP (TNBS+SASP, 100 mg/kg b.wt, intragastric, dissolved in saline), Sal B(L) (TNBS+Sal B(L), 20 mg/kg b.wt, intragastric, dissolved in saline), and Sal B(H) (TNBS+Sal B(H), 80 mg/kg b.wt, intragastric, dissolved in saline), respectively. IBD recurrence in different groups was observed after intraperitoneal (*ip*) injection of recombinant IL-1β for 48 h (from day 15 to 16; Figure 1).

### Colitis induced by TNBS and recurrence of colitis with intraperitoneal injection of IL-1β

The rat colitis model was induced according to the previous reports with slight modification (Morris et al., 1989; Aube et al., 1999). Briefly, a catheter was inserted to the level of the splenic flexure (8 cm proximal to the anal verge) after a fasting period of 24 h with free access to drinking water, under urethane anesthesia. TNBS dissolved in ethanol (40%V/V) was then infused to the colon at a dose of 125 mg/kg. The sham group rats were similarly treated but infused with 1 mL ethanol. Rats were allowed to eat and drink *ad libitum* 1 h later. Biochemical studies were then performed using full thickness of intestinal wall in the distal colonic samples. The study of colitis recurrence was conducted according to the methods reported previously with modifications (Watanabe et al., 1997; Young Oh et al., 2008). Briefly, the recurrence of colitis after SASP or Sal B treatment was induced by giving rats recombinant human IL-1β (150 μg/kg) intraperitoneally for 48 h from day 15. At day 17, the colitis symptoms were checked to test weather colitis recurrence was found.

### Assessment of inflammation

Inflammatory factors of colitis model including animal body weights, diarrhea incidence, and total food intake were recorded everyday. Colon macroscopically visible damage was measured using a 0–10 scale (Orsi et al., 2014). According to the morphological criteria described previously, routine hematoxylin and eosin (HE)-stained colon section was measured to represent the degree of inflammation (Cooper et al., 1993; Ghia et al., 2008; Schwanke et al., 2013). After being sacrificed, the colon tissues of the animals were embedded in paraffin and fixed in 10% formalin for at least 24 h. The samples were then cut into 5 mm pieces and fixed on slides according to the routine procedure. Colon sections were HE-stained (or used for other immunohistochemistry studies). Then, the samples were analyzed by light microscopy (Nikon Eclipse TE2000-U, Nikon Corp., Tokyo, Japan).

### Evaluation of epithelial barrier function

According to the previous literatures, intestinal permeability (barrier function) was measured *in vivo* as follows. Rats were fasted but free to water for 3 h. Then, rats received gavage treatment of 80 mg/mL fluorescein isothiocyanate-4 kD dextran (FD-4) (Sigma, St. Louis, MO, USA). After 1 and 3 h later, serum was harvested and measured using a Synergy HT plate reader (BioTek, Winooski, VT, USA), respectively. Intestinal permeability was represented with enhanced serum recovery of FD-4.

Intestinal barrier function *in vitro* was studied by measurement of transepithelial electrical resistance (TER). Induced TER is parameter of disturbed intestinal barrier function (Smith and Clark, 2010; O'Malley et al., 2012). TER of filter-grown Caco-2 intestinal monolayers was measured using an epithelial voltohmmeter. Caco-2 cells (4 × 10^5^) were seeded in the upper chamber of a transwell filter. TER of Caco-2-plated filters was measured daily. After TER reached to a steady state (always 540 ± 12 Ω/cm^2^) 5 days later, Caco-2-plated filters could be used to establish barrier function model. Barrier dysfunction cellular model was established in Caco-2 monolayers exposed with IL-1β (10 ng/mL). TER always decreased to 340 ± 9 Ω/cm^2^ after IL-β treatment (Al-Sadi and Ma, 2007). Sal B (20, 40, 80 μM) could reverse the decreased TER level to (350 ± 10.5–450 ± 9 Ω/cm^2^). The relative TER in Sal B treatment group was calculated as a percentage of IL-1β control group and TER in IL-1β control group was set to 100%. TER determination was repeated for at least three times.

### Quantitative RT-PCR

As previously described, total RNA was isolated with RNeasy mini kit (Qiagen) (Chang-Qing et al., 2009). RNA samples with an A260/280 ratio between 1.9 and 2.1 were used. A TaqMan miRNA Reverse Transcription Kit was used for reverse transcription assay. A TaqMan miRNA assay Kit (GenePharma Corp.) and Applied Biosystems 7500 FAST System (Applied Biosystems, Foster City, CA, USA) were used for mature miRNA measurement. U6 was used as an internal control. The levels of miRNA were determined using the formula 2^−ΔΔ^Ct.

### Dual luciferase reporter assays

Plasmid DNA (wt-Luc-MLCK, mut-Luc-MLCK, or control vector) and antago-miR-1 or the antago-miR negative control were co-transfected into Caco-2 cells. Cells were incubated with 20, 40, and 80 μM Sal B 24 h after transfection, respectively. After 24 h, reporter assays were performed. Dual-Light Chemiluminescent Reporter Gene Assay System (Berthold, Germany) was used to measure luciferase activity, which then was normalized to Renilla luciferase activity.

### Measurement of ROS production

The ROS content in RAW264.7 cells was measured using flow cytometry according to previous reports (Abdul-Sater et al., 2010). Briefly, RAW264.7 cells were plated in phenol red-free DMEM containing 10% FBS and then infected and/or treated for the indicated times. Cells were detached using TrypLE^TM^ Express, loaded with 2.5 μM DCF in PBS for 15 min at 37°C, washed with PBS, resuspended in growth medium, and finally analyzed by flow cytometry with a Guava EasyCyte system with a 15-mW argon ion laser at 488 nm. Fluorescence was measured on the FL1 (green) channel, gating only on live cells. RAW264.7 cells were treated with IL-1β or Sal B. Non-treated RAW264.7 cells served as normal control.

### Western blot analysis

Total Protein Extraction Kit (KeyGEN Bio TECH, Nanjing, China) was used for total protein isolation from full thickness of intestinal wall. BCA Protein Assay Kit (Beyotime Institute of Biotechnology, Haimen, China) was used for protein concentration determination. The blots on PVDF membrane were respectively probed with corresponding protein antibodies including MLCK antibody [1:1000; Abcam (Hong Kong) Ltd.], claudin-2 antibody (1:800; Santa Cruz Biotechnology Co. Ltd., Shanghai, China), iNOS antibody [1:1000; Abcam (Hong Kong) Ltd.], COX-2 antibody [1:1000; Abcam (Hong Kong) Ltd.], and NF-KappaB-p65 antibody [1:1000; Abcam (Hong Kong) Ltd.], respectively. Multi Spectral imaging system (UVP, Cambridge, UK) was used to detect and quantify the bands for special protein determination.

### Statistical analysis

In this study, data from rats in sham group was studied compared with that in TNBS group using One-Way ANOVA firstly, and then the data from rats in saline+TNBS, Sal B+TNBS, and SASP+TNBS group was studied compared with that in TNBS group using pair wise comparison of each group to each group in One-Way ANOVA. Data were expressed as the mean ± *S.D*. All experiments were repeated for at least three times or in multiple animals as indicated. ^**^p < 0.05 was considered as significantly different.

## Results

### Salvianolic acid B-induced modulations on oxidative stress and epithelial barrier function in normal rats

The cytotoxicity of Sal B was studied in mouse macrophage cell line RAW264.7. Sal B in the dose range of 5–1280 μM showed almost no cytotoxicity effects (data not shown). Based on these pre-experiments and preliminary results reported by other researchers, 20 mg/kg (low dose) and 80 mg/kg (high dose) Sal B were selected for animal experiments.

**Figure 2:**
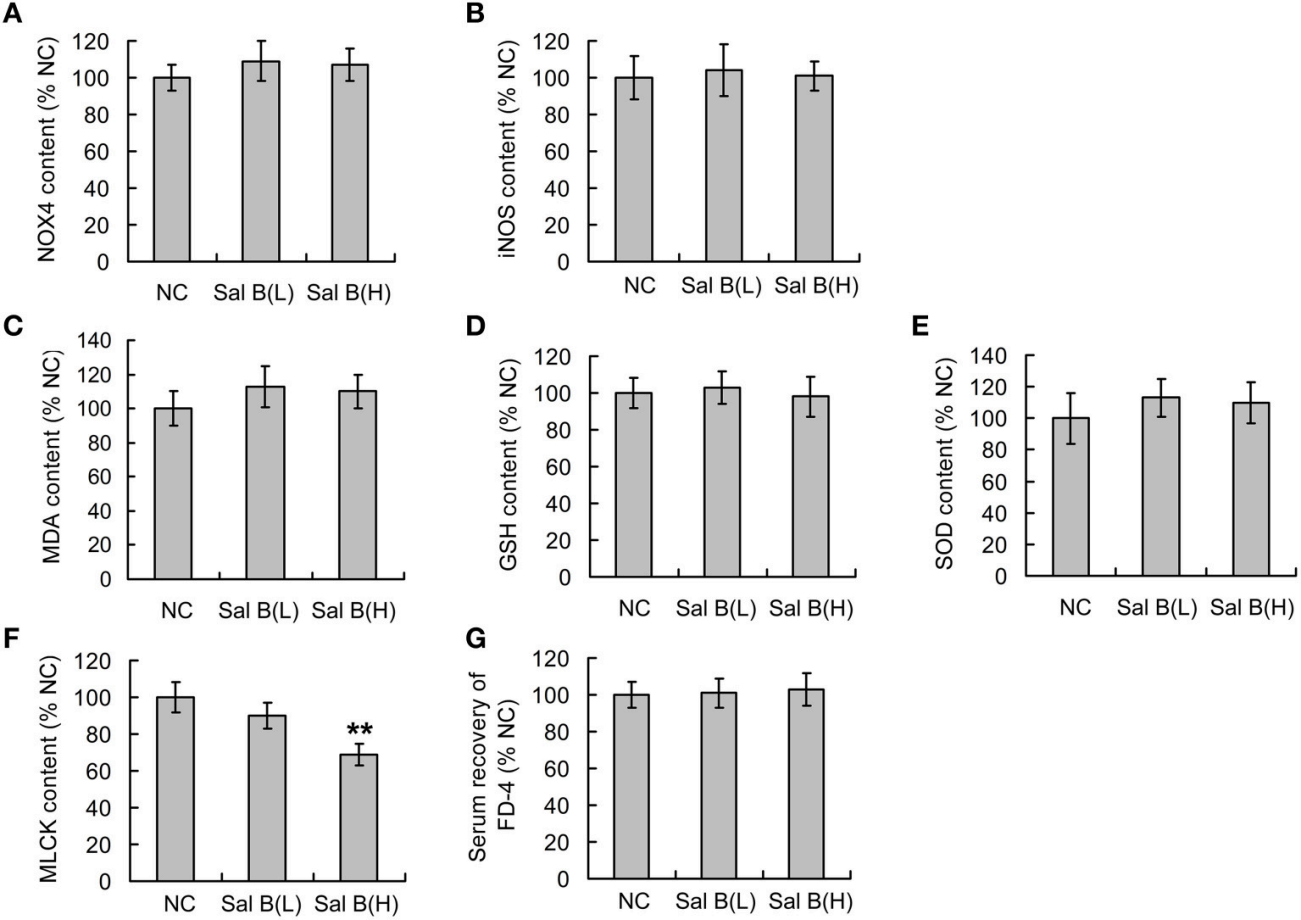
**Salvianolic acid B (Sal B) induced modulations on oxidative stress and epithelial barrier function in normal rats**. After gavage administration of Sal B once a day for seven consecutive days, the contents of **(A)** NADPH oxidase 4 (NOX4), **(B)** inducible nitric oxide synthase (iNOS), **(C)** malondialdehyde (MDA), **(D)** glutathione (GSH), **(E)** superoxide dismutases (SOD), and **(F)** long myosin light chain kinase (MLCK) from colon tissue in normal control (NC) rats were observed using ELISA methods; **(G)** the effects of Sal B on the serum recovery of FD-4 was also observed. Data are expressed as the mean ± *SD*. ^**^P < 0.01, compared with the NC group (*n* = 6). Data in the NC group is set to 100%; other data are the relative values compared with those in the NC group. Sal B(L), 20 mg/kg Sal B; Sal B(H), 80 mg/kg Sal B.

The effects of Sal B on oxidative stress and epithelial barrier function in normal rats were respectively studied before the analysis of Sal B induced modulation on those in colitis rats. ELISA results showed that the low dose and high dose of Sal B did not affect the expression of NOX4, iNOS, MDA, GSH, and SOD (Figures 2A–E) from colon tissue, while the high dose of Sal B decreased the expression of long MLCK (Figure 2F) from colon tissue. The serum recovery of FD-4 in normal rats was not changed after Sal B treatment, which indicates that Sal B does not affect the intestinal barrier function of normal rats (Figure 2G).

### General conditions observed in rats

Rats in the phosphate buffered saline (PBS) group (intracolonic administration of PBS) woke up about 1 h after anesthesia with no abnormal inflammatory symptoms found, compared to normal control rats with no operation. Rats in the ethanol group (intracolonic administration of ethanol) woke up about 1 h after anesthesia and symptoms presented within the first 2 days, including shapeless stool and loss of appetite. The symptoms disappeared after 3 days of ethanol treatment. Three days after the operation, HE-staining results of colonic tissue showed no difference between ethanol/PBS treated rats and normal control rats (data not shown). In the following study, the ethanol group was set as the sham group to study the effects of Sal B induced modulation in colitis rats.

The rats in the TNBS group woke up about 2 h after anesthesia with symptoms including shapeless stool, increased stool frequency, outflow of red or dark red liquid from the anus, positive fecal occult blood test, and loss of appetite; the symptoms peaked on the 3rd day and lasted for about 7 days. After 7 days of TNBS induction, the shapeless stools were also seen in the TNBS, Sal B, and SASP treated groups but fecal occult blood test was negative. No rats, three rats, two rats, two rats, and two rats died in the ethanol, TNBS, SASP, low, and high dose Sal B groups, respectively.

### Salvianolic acid B treatment ameliorated colitis symptoms

**Figure 3:**
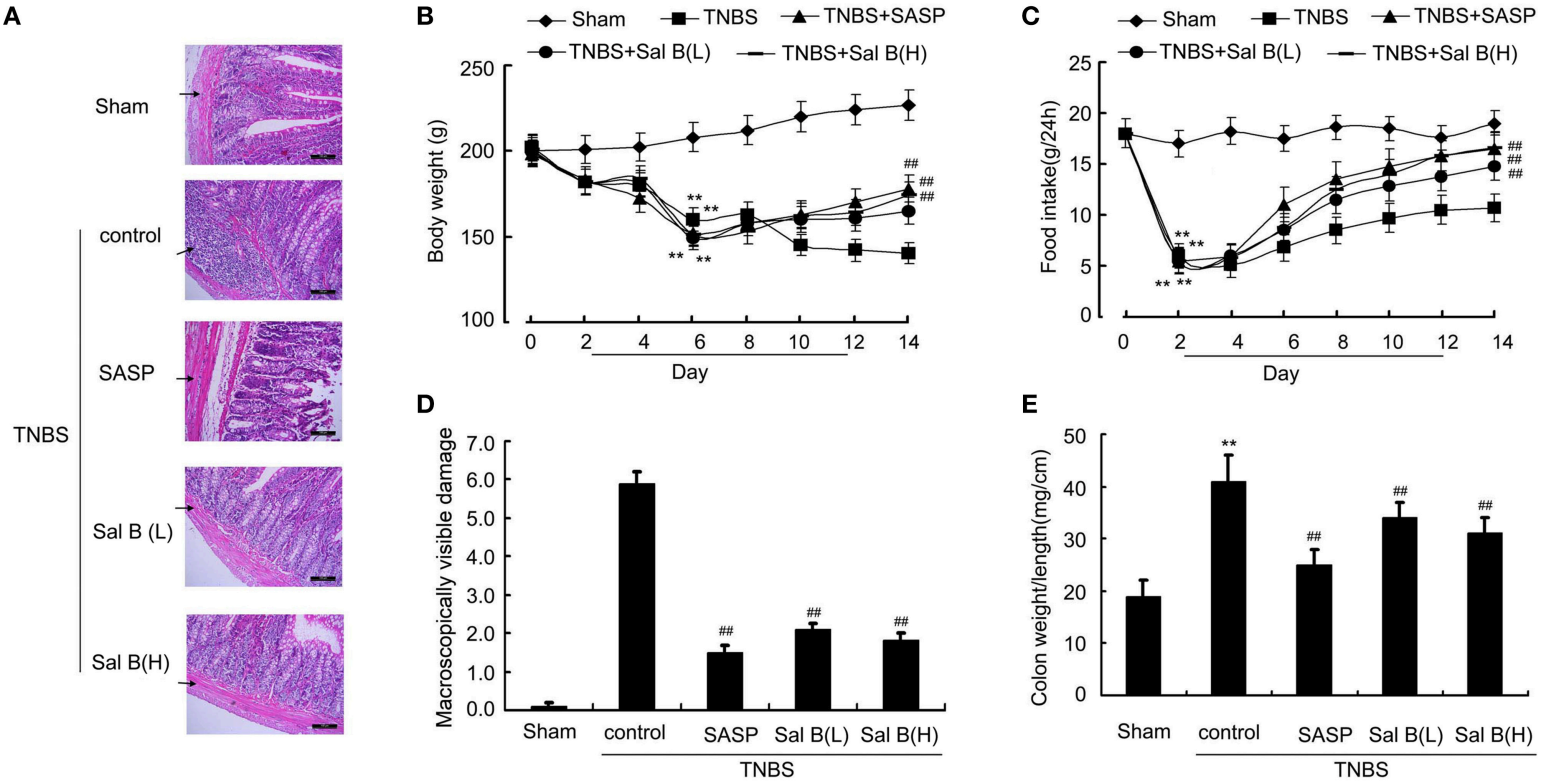
**Salvianolic acid B (Sal B) ameliorated colitis symptoms. (A)** Hematoxylin and eosin (HE)- staining analysis of rat colonic tissue. Effects of Sal B on **(B)** body weight, **(C)** food intake, **(D)** macroscopically visible damage, and **(E)** colon weight-to-length ratio in colitis rats. Data are expressed as the mean ± *SD*. ^**^P < 0.01 compared with the sham group (*n* = 7); ^##^*P* < 0.01 compared with the TNBS control group (*n* = 7). SASP, sulfasalazine; Sal B(L), 20 mg/kg Sal B; Sal B(H), 80 mg/kg Sal B.

Significant lower body weight and food intake were observed in the sham group after the induction of inflammation (Figures 3B,C). Successful establishment of colitis was confirmed with biochemical and macroscopic analysis. Increased macroscopically visible damage and colon weight-to-length ratio were observed after TNBS treatment (Figures 3D,E). Sal B (20 and 80 mg/kg) and SASP significantly ameliorated the colitis symptoms induced by TNBS, which included an increase in body weight and food intake, and a decrease in macroscopically visible damage and colon weight-to-length ratio (Figures 3A–E). Together, these data show that Sal B can suppress inflammation in the TNBS-induced colitis model.

### Salvianolic acid B reduced neutrophil infiltration and cytokine profiles

**Figure 4:**
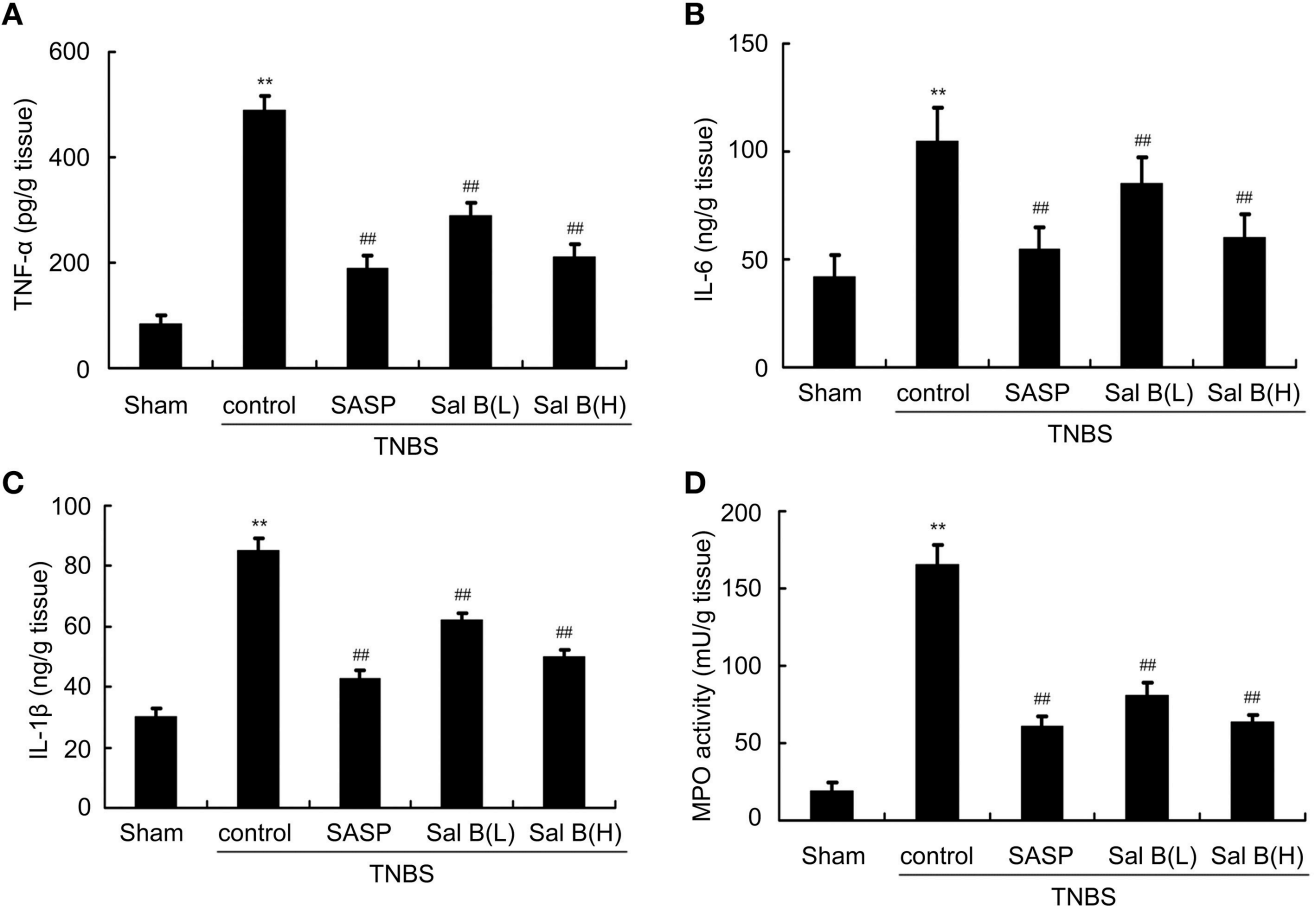
**Salvianolic acid B (Sal B) reduced neutrophil infiltration and cytokine profiles**. ELISA results of Sal B-induced effects on proinflammatory cytokine **(A)** TNF-α, **(B)** IL-6, **(C)** IL-1β, and **(D)** myeloperoxidase (MPO) from rat colon tissue. Data are expressed as the mean ± *SD*; ^**^P < 0.01 compared with the sham group; ^##^*P* < 0.01 compared with the TNBS control group (*n* = 7). SASP, sulfasalazine; Sal B(L), 20 mg/kg Sal B; Sal B(H), 80 mg/kg Sal B.

Initiation of colonic inflammation often associates with the enhancement of neutrophil infiltration and levels of pro-inflammatory cytokines (Scull et al., 2010; Bian et al., 2012; Lin et al., 2016). Neutrophil infiltration into the damaged tissue is represented by MPO activity (Piegeler et al., 2014). Pro-inflammatory cytokines (TNF-α, IL-1β, and IL-6) levels and MPO activity from colitis rat colon tissue were significantly increased compared to those in sham group (Figures 4A–D). Increased MPO activity and changes in cytokine levels were reversed by SASP and Sal B (20 or 80 mg/kg) treatment.

### Salvianolic acid B restored impaired barrier function *in vivo*

**Figure 5:**
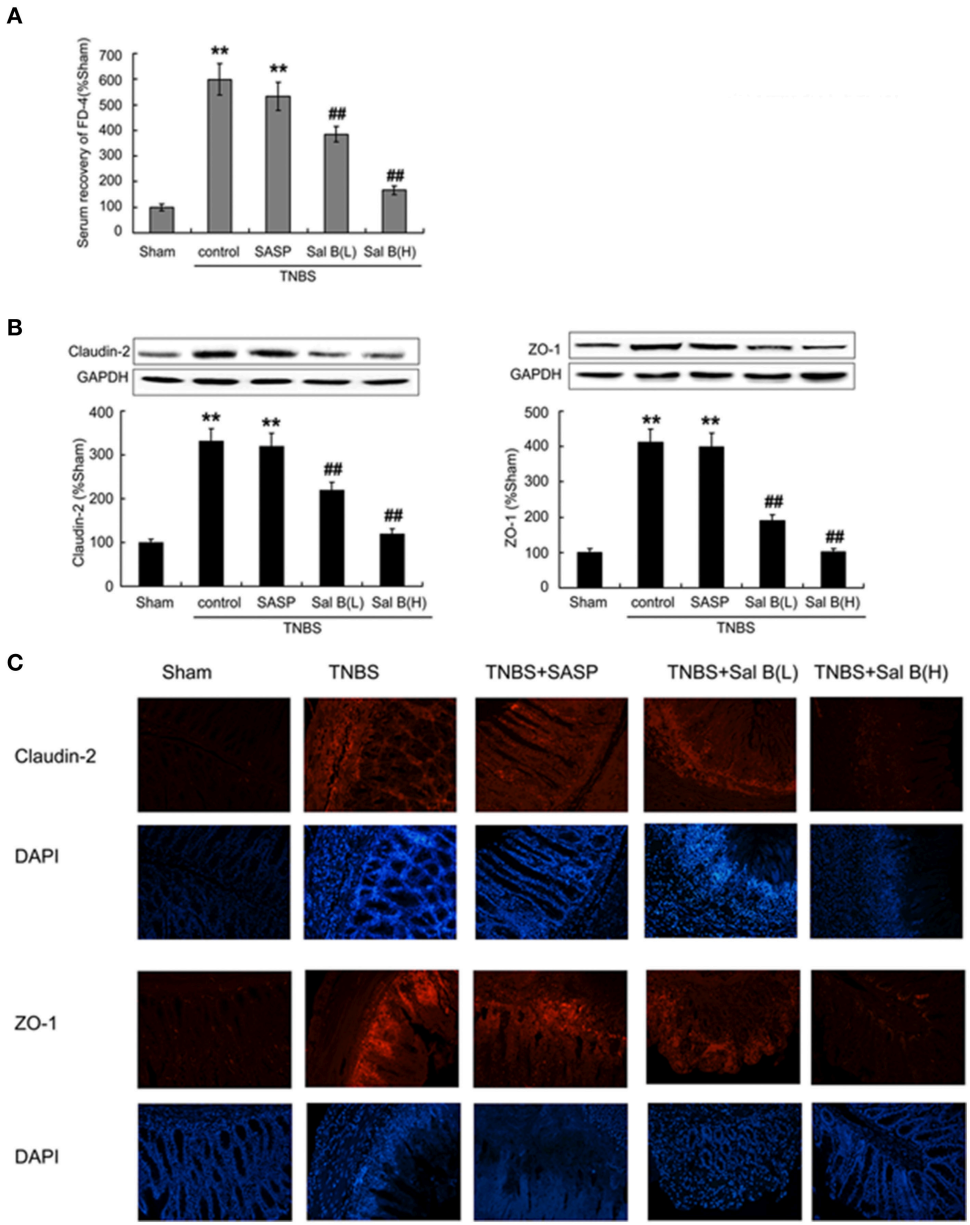
**Salvianolic acid B (Sal B) restored barrier function through decreasing intestinal permeability. (A)** Effects of Sal B on the serum recovery of FD-4. Effects of Sal B on tight junction protein expression of claudin-2 and zona occludens ZO-1 from colon tissue using western blotting **(B)** and immunofluorescence technique **(C)**. Data are expressed as the mean ± *SD*; ^**^P < 0.01 compared with the sham group; ^##^*P* < 0.01 compared with the TNBS control group (*n* = 7). Data in the sham group is set to 100%; other data are the relative values compared with those in the sham group. SASP, sulfasalazine; Sal B(L), 20 mg/kg Sal B; Sal B(H), 80 mg/kg Sal B.

A significant increase in serum recovery of FD-4 in the TNBS control group compared with the sham group was found after 14 days of the inflammation induction (Figure 5A), which was accompanied with the increase of tight junction protein expressions like claudin-2 and zona occludens (ZO-1) in the TNBS control group (Figure 5B). Immunofluorescence results of claudin-2 and ZO-1 was also enhanced in TNBS control group, compared with in normal control (Figure 5C). These results indicate that the intestinal barrier dysfunction was not restored, although HE-staining results showed that the epithelium was almost intact in the TNBS control group. After 13 days of Sal B treatment, all these changes were reversed. The high expressions were significantly decreased by Sal B but not SASP.

### MiR-1 activation and MLCK inactivation was involved in sal B-induced restoration of barrier function

**Figure 6:**
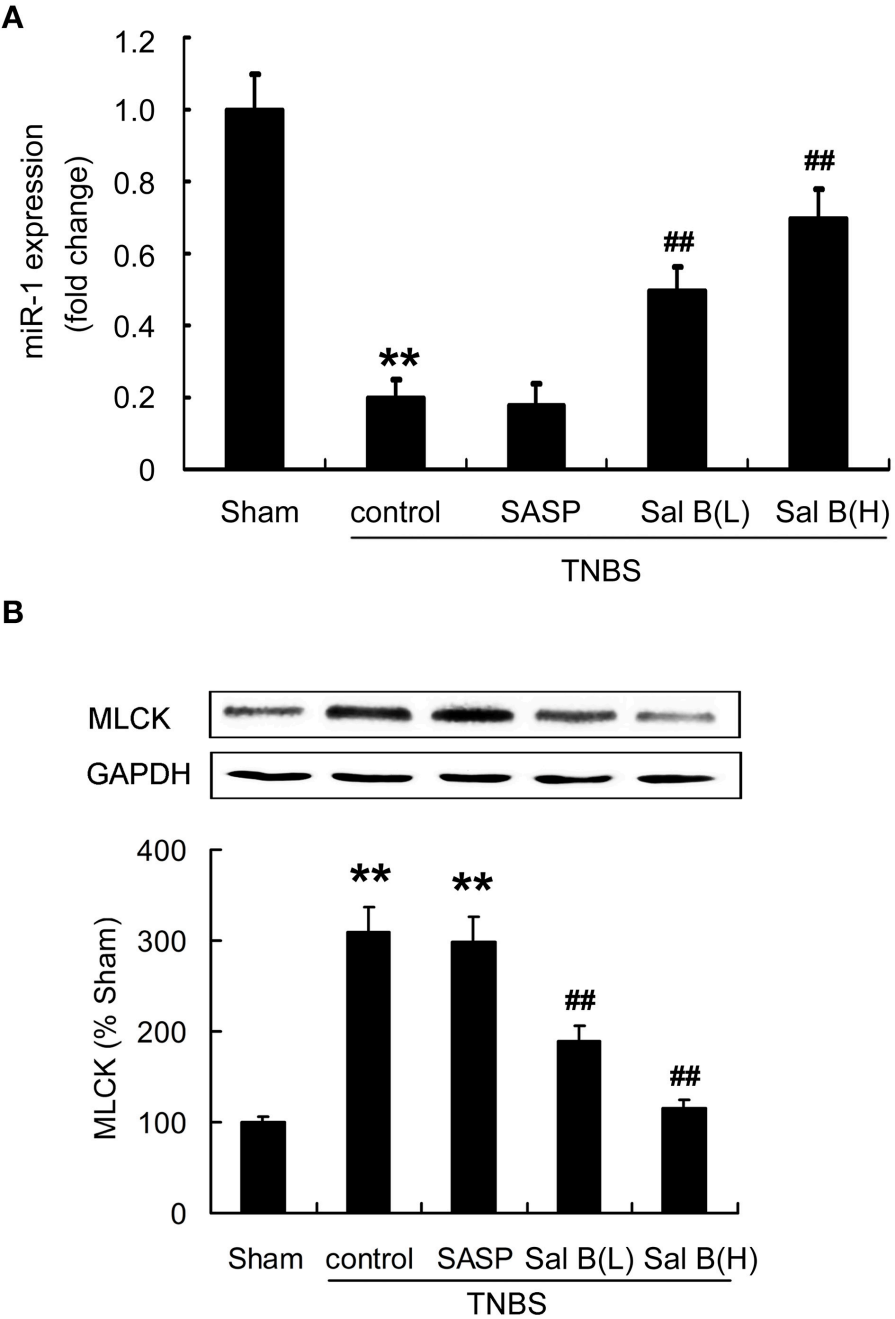
**Salvianolic acid B (Sal B) reversed the altered microRNA-1(miR-1) and MLCK expression**. Effects of Sal B on the expression of **(A)** miR-1 and **(B)** MLCK in rat colitis model was measured. Data are expressed as the mean ± SD; ^**^P < 0.01 compared with the sham group; ^##^*P* < 0.01 compared with the TNBS control group (*n* = 7). Data in the sham group is set to 100%; other data are the relative values compared with those in the sham group. SASP, sulfasalazine; Sal B(L), 20 mg/kg Sal B; Sal B(H), 80 mg/kg Sal B.

To test our hypothesis that Sal B-induced restoration of barrier function is mediated by miR-1/MLCK pathway, we measured the effects of Sal B on miR-1 and MLCK expressions in colitis rat model. As shown in Figure 6, miR-1 expression was decreased in TNBS model group, while MLCK protein content was significantly increased. Sal B treatment reversed the changes of expression of miR-1 and MLCK respectively, however, SASP did not alter miR-1 and MLCK expression in rat colitis model (Figure 6).

### MiR-1 mediated sal B-induced restoration effects by targeting of MLCK

**Figure 7:**
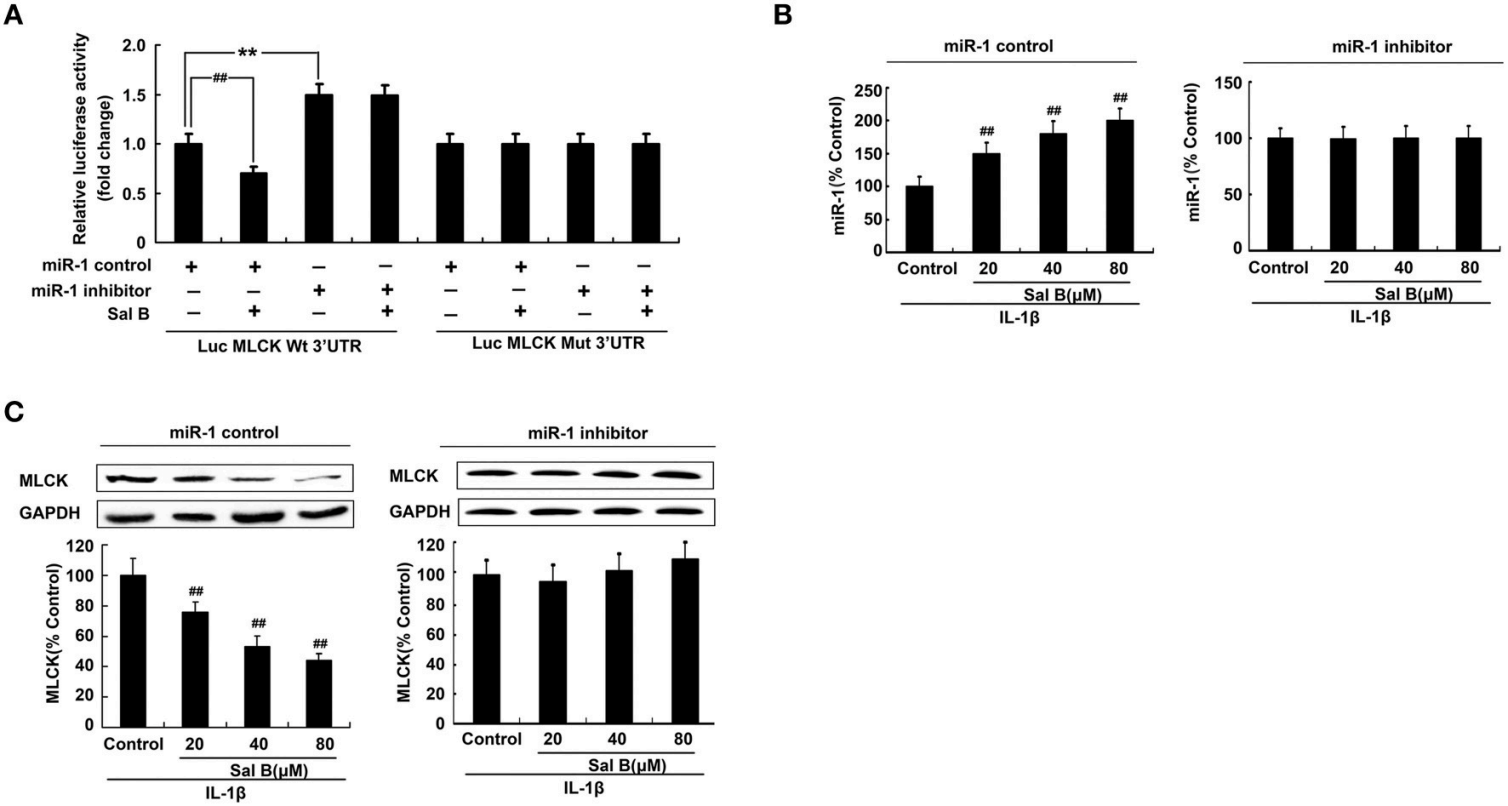
**MicroRNA-1(miR-1) mediated Sal B-induced restoration effects by targeting of MLCK. (A)** Sal B abrogates the increase in MLCK following miR-1 repressed. **(B–C)** Sal B abrogates the increase in MLCK following miR-1 repressed. The data are presented as the mean ± *SD*. (*n* = 3). ^**^P < 0.01 vs. the antago-miR control group, ^##^*P* < 0.01 vs. miR-1 or MLCK before Sal B treatment respectively.

Antago-miR-1 and luciferase reporter plasmids containing the miR-1-MLCK response element (wt-Luc-MLCK) or a mutant miR-1-MLCK response element (mut-Luc-MLCK) were co-transfected to Caco-2 cells in the presence or absence of Sal B, respectively. Luciferase activity was significantly decreased by Sal B treatment and this effect was significantly blocked by miR-1 inhibitor. However, these effects were not observed with the mutated MLCK-3′-UTR (Figure 7A).

**Figure 8:**
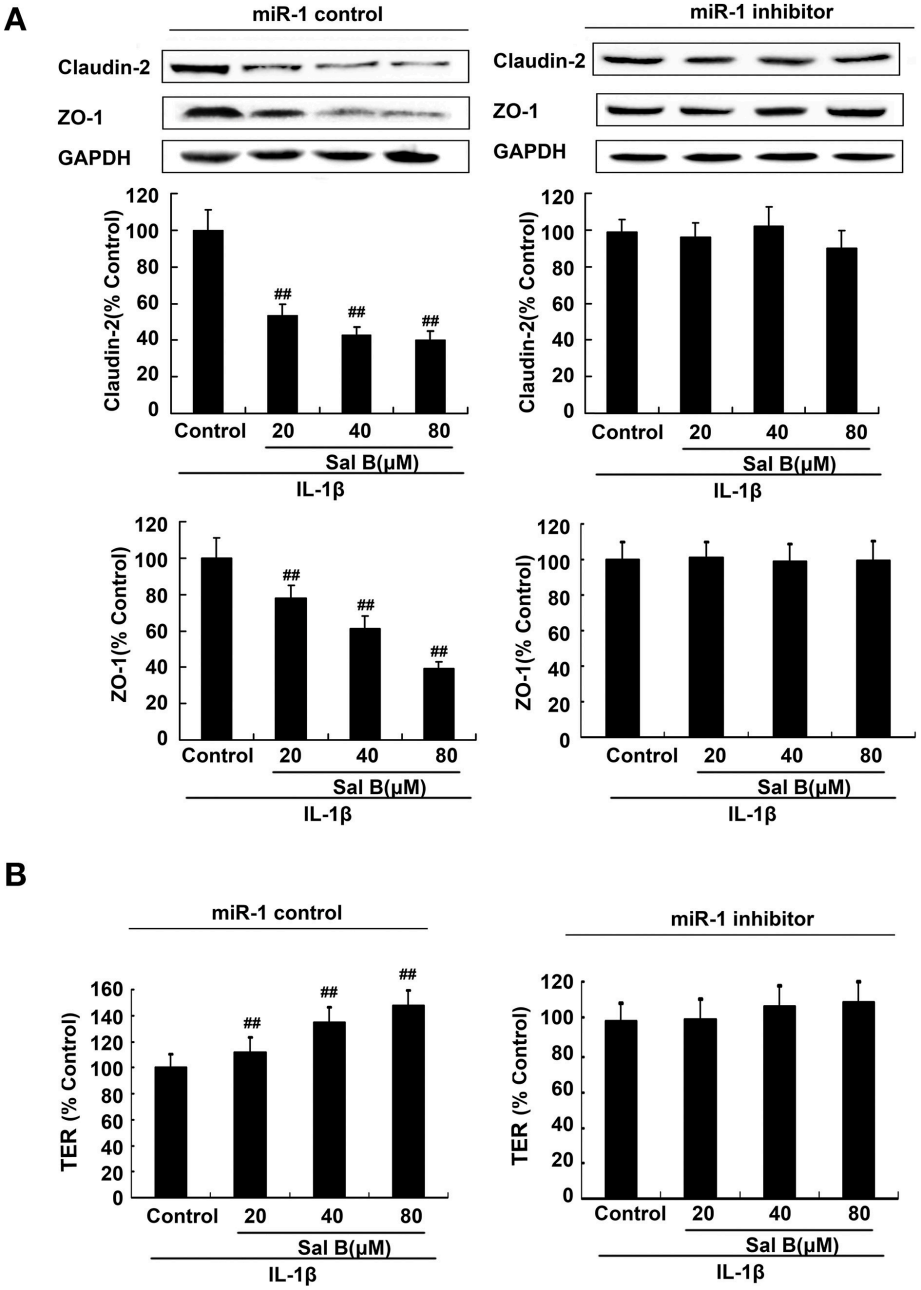
**Sal B-induced restoration effects was abolished by microRNA-1(miR-1) inhibition *in vitro*. (A)** Sal B-induced inhibition on tight junction proteins claudin-2 and zona occludens (ZO-1) contents and **(B)** stimulation on transepithelial electrical resistance (TER) levels were abolished in the present of miR-1 inhibition. The data are presented as the mean ± *SD*. (*n* = 3). ^##^*P* < 0.01 vs. tight junction proteins or TER levels before Sal B treatment respectively.

To further elucidate the cellular effects of Sal B on MLCK, Caco-2 cells with antago-miR-1 were transfected in the presence or absence of Sal B, respectively. And the expression levels of miR-1 and MLCK were also determined. MiR-1 expression in Caco-2 cells was significantly increased by Sal B incubation and this effect could be blocked by cell transfection with miR-1 inhibitor (Figure 7B). The high expression of MLCK in Caco-2 cells induced by IL-1β was significantly decreased by Sal B treatment, however, after cell transfection with miR-1 inhibitor, the inhibitory effect of Sal B on MLCK expression was significantly blocked (Figure 7C). Sal B decreased the enhanced protein levels of claudin-2 and ZO-1 triggered by IL-1β. Sal B also reversed the reduced TER levels induced by IL-1β (Figure 8). These effects of Sal B on claudin-2, ZO-1 expression and TER changes were also significantly blocked by miR-1 inhibition. Thus, we concluded that Sal B targeted miR-1, induced downregulation of MLCK and finally resulted in restoration of defective tight junction barrier function in rat colitis model.

### Salvianolic acid B increased the antioxidant capacity *in vivo* and *in vitro*

**Figure 9:**
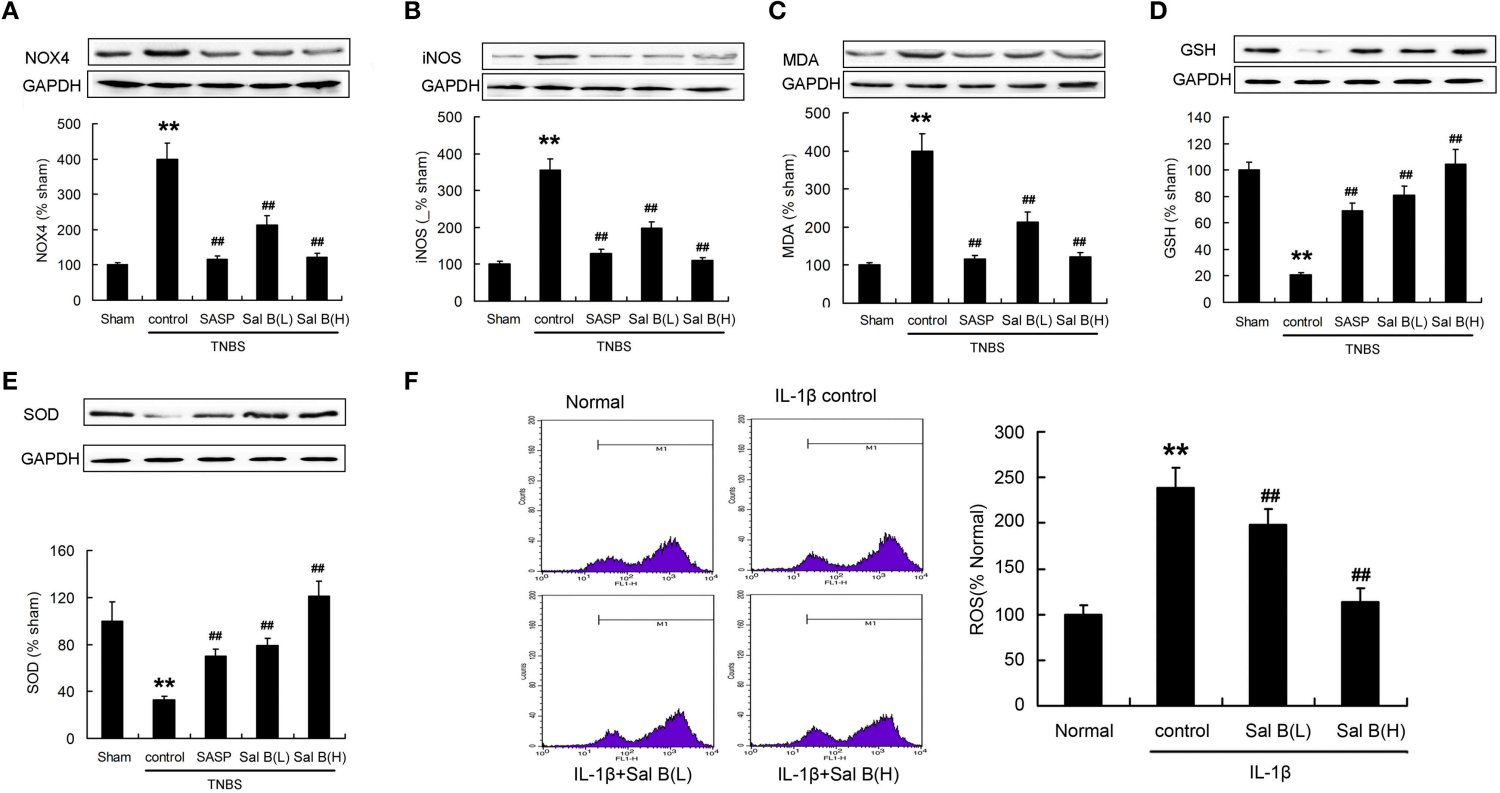
**Salvianolic acid B (Sal B) increased the antioxidant capacity**. Effects of Sal B on protein expression of **(A)** NADPH oxidase 4 (NOX4), **(B)** inducible nitric oxide synthase (iNOS), **(C)** malondialdehyde (MDA), **(D)** glutathione (GSH), **(E)** superoxide dismutases (SOD) in colon tissue; **(F)** Effects of Sal B on reactive oxygen species (ROS) production in RAW264.7 cells. Data are expressed as the mean ± *SD*; ^**^P < 0.01 compared with the sham (colon tissue) or normal (cell) group; ^##^*P* < 0.01 compared with the TNBS (IL-1β) control group (*n* = 7). Data in the normal or sham group is set to 100%; other data are the relative values compared with those in the sham group. SASP, sulfasalazine; Sal B (L), 20 mg/kg Sal B; Sal B (H), 80 mg/kg Sal B.

Protein expressions of NOX4, iNOS, and MDA were significantly increased, while GSH and SOD were significantly decreased compared to those in the sham group after the induction of inflammation (Figures 9A–E). ROS levels determined using flow cytometry was increased after IL-1β treatment *in vitro* (Figure 9F). These data show that the antioxidant system in colitis model may be destroyed. Sal B treatment increased the antioxidant capacity, which decreased the protein expressions of NOX4, iNOS, MDA, and ROS levels, and increased the protein expression of GSH and SOD (Figures 9A–F).

### Analysis of colitis recurrence induced by IL-1β after Sal B treatment

**Figure 10:**
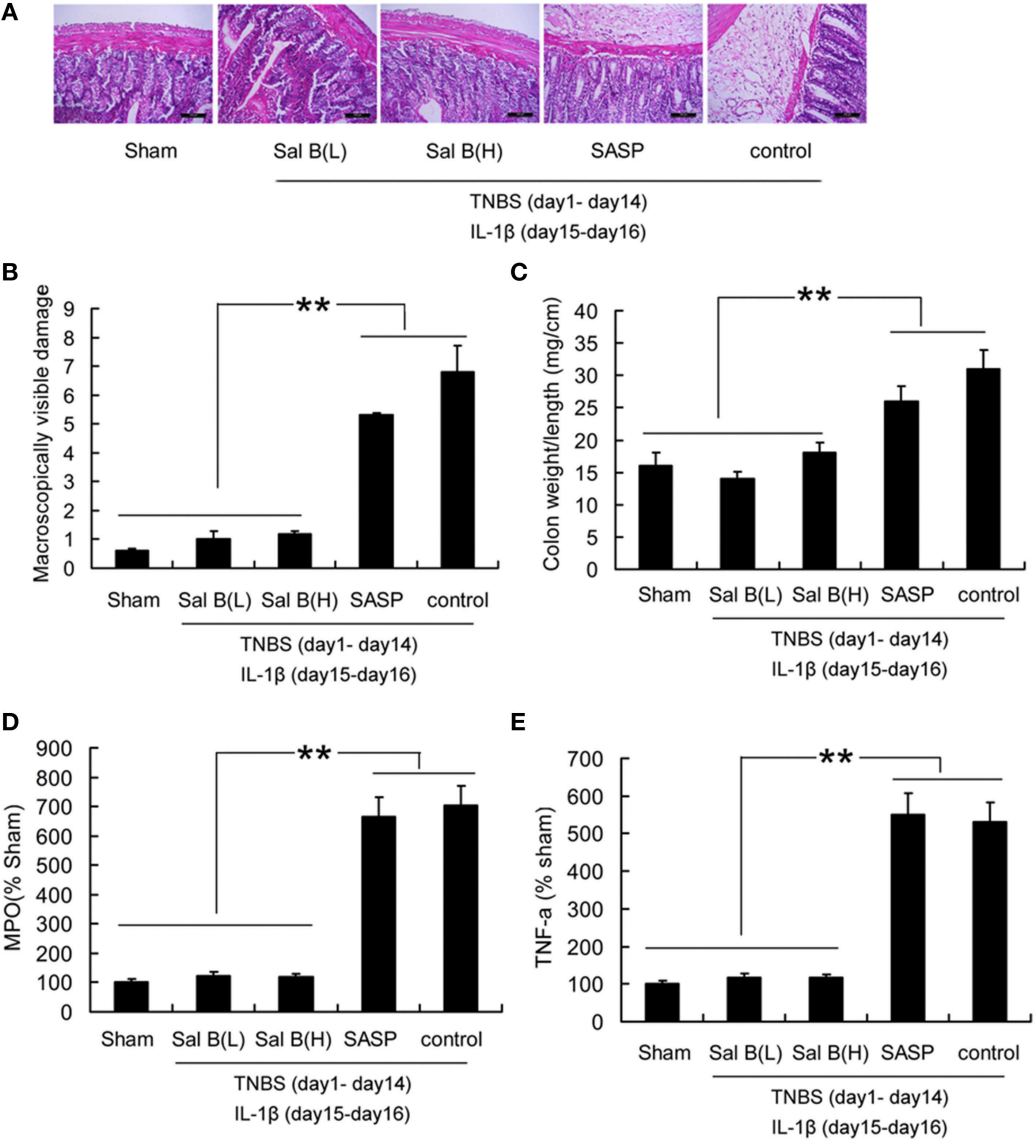
**Analysis of colitis recurrence induced by IL-1β (10 ng/mL). (A)** HE-staining analysis of colitis recurrence induced by IL-1β (10 ng/mL) after SASP or Sal B treatment in rat colonic tissue. Effects of Sal B on **(B)** macroscopically visible damage, **(C)** colon weight-to-length ratio, **(D)** myeloperoxidase (MPO), **(E)** proinflammatory cytokine TNF-α. Data are expressed as the mean ± *SD*; ^**^P < 0.01 compared with as indicated (*n* = 10). Data in the sham group is set to 100%; other data are relative values. SASP, sulfasalazine; Sal B(L), 20 mg/kg Sal B; Sal B(H), 80 mg/kg Sal B.

After 13 days of treatment by SASP or Sal B, the symptoms in colitis rats were significantly alleviated, this can be proved by morphological or histological checks (Figure 3). However, the defective tight junction barrier function in the TNBS group was not significantly affected by SASP (Figure 5). Recurrence analysis was carried out in order to study the potential of Sal B for treatment of colitis. As shown in Figure 10, *ip* administration of IL-1β at day 15 after TNBS induced colitis led to 90% of colitis recurrence in the SASP treated group, 10% in the Sal B(L) treated group, and 20% in the Sal B(H) treated group. Significantly attenuated levels of inflammatory cytokines TNF-α, MPO, macroscopically visible damage score, and colon weight-to-length ratio were responsible for the resistance to colitis recurrence in Sal B treated group (Figure 10).

## Discussion

In the present study, the effects of Sal B on colitis were assessed morphologically, histologically and pharmacologically in a TNBS-induced rat colitis model. Sal B significantly ameliorated the symptoms induced by TNBS, including decreasing the gross damage score, MPO activity, and cytokine profiles compared with those in the TNBS control group. The enhanced intestinal epithelial permeability in the TNBS control group was decreased by Sal B. Both SASP and Sal B alleviated TNBS induced colitis. Sal B restored the defective tight junction barrier function. However, SASP, as a well-known anti-inflammation drug, exerted no effects on disturbed tight junction barrier function. The defective tight junction barrier function leads to increased intestinal permeability and finally aggravates IBD (Su et al., 2013). Our results also showed that Sal B-induced restoration of defective tight junction barrier function has a protective effect against IL-1β-induced colitis recurrence. Therefore, Sal B induced restoration of impaired tight barrier function play an important role in the treatment of colitis as well as the colitis recurrence.

MicroRNAs play an important role in a wide range of pathophysiological cellular processes (Chen, 2007; Bogatcheva et al., 2011). Aberrant expression of miRNAs, which have been proved to be novel therapeutic targets for human diseases, is involved in the pathophysiology of a number of human diseases including inflammation, cancer, cardiovascular diseases (Milenkovic et al., 2013; Tomé-Carneiro et al., 2013). In this study, we found that Sal B targeted miR-1, induced downregulation of MLCK and finally resulted in restoration of defective tight junction barrier function in rat colitis model. Therefore, miR-1 may alleviate colitis symptoms through restoring the impaired barrier function. Epithelial permeability in the TNBS control group was significantly increased compared to the sham group; and the increase could be significantly decreased by Sal B treatment, which was accompanied with a decrease in the expression of tight junction proteins including claudin-2 and ZO-1 (Poritz et al., 2007; Bai et al., 2008; O'Driscoll et al., 2010). After 14 days of the inflammation induction operation, HE-staining results showed that the epithelium was almost intact, a significantly increased serum recovery of FD-4 in the TNBS control group was also found. The tight junction proteins levels were also significantly increased in the TNBS control group compared to those in the ethanol group, which proves that intestinal barrier function with intact epithelium is factually defined by epithelial permeability.

The colitis model induced by TNBS (diluted in 50% ethanol) is useful in following temporal changes of inflammation visually and histologically, and is appropriate for the study of inflammatory responses, as well as epithelial alterations upon mucosal damage (González et al., 2009; Lejeune et al., 2010; Brenna et al., 2013). In the SASP treated group, the inflammatory responses were significantly decreased compared with those in the TNBS control group; however, the high epithelial permeability was almost unchanged. After intestinal resection there is a high symptomatic recurrence rate in the first year after surgery (Lochs et al., 2000; Rutgeerts, 2002). Anti-inflammation therapy (SASP, other 5-aminosalicylic acid formulations or probiotics) has limited value in the prophylaxis of colitis recurrence (Lochs et al., 2000; Prantera et al., 2002). We proposed that Sal B induced restoration of epithelial barrier function may supply some new treatment alternatives for the prophylaxis of colitis recurrence.

In the case of radical scavenging activity, results in the present study showed that the content of cellular ROS, colonic nitric oxide (NO) and the cytokine profiles including TNF-α, IL-6, and IL-1β was significantly decreased by gavage administration of Sal B. The infiltration of inflammatory cells is regarded as a trigger of free radical release (Bhaskar et al., 1995), which then attack cellular structure through the inhibition of endogenous defense systems (Pham-Huy et al., 2008; Wu et al., 2013). In this study, TNBS-induced oxidative stress was indicated by the elevation of NOX4, inducible nitric oxide synthase (iNOS), MDA level, and overwhelming GSH and SOD levels, finally led to the increased accumulation of ROS and NO. The serious imbalance between the production of free radicals and antioxidant defense in colitis could be significantly reversed by Sal B. HE-staining results showed that the alterations in the TNBS control group, including epithelial necrosis, impaired mucosa involving submucosa with hyperemia and edema, and ulceration accompanied with numerous inflammatory cell infiltrations, were alleviated by gavage administration of Sal B.

In conclusion, Sal B could restore barrier function, which was mediated by the activation of miR-1 and the subsequent MLCK inactivation. The restoration effect of epithelial barrier function is also beneficial for the prophylaxis of colitis recurrence. Inhibition of oxidative stress and reduction of cytokine secretion is involved in Sal B exerted anti-inflammatory effect. Such data may provide some novel insights that might help to develop more treatment alternatives for IBD.

## Author contributions

Conceived and designed the experiments: DC, HG, and YX. Performed the experiments: HG, YX, JW, and HC. Analyzed and interpreted the data: JW and HC. Drafted the paper and revised it critically for important intellectual content: DC, HG, YX, JW, and HC. The manuscript has been approved by all the authors.

### Conflict of interest statement

The authors declare that the research was conducted in the absence of any commercial or financial relationships that could be construed as a potential conflict of interest.
